# Predicting the most appropriate wood biomass for selected industrial applications: comparison of wood, pulping, and enzymatic treatments using fluorescent-tagged carbohydrate-binding modules

**DOI:** 10.1186/s13068-017-0980-0

**Published:** 2017-12-06

**Authors:** Pierre-Louis Bombeck, Vinay Khatri, Fatma Meddeb-Mouelhi, Daniel Montplaisir, Aurore Richel, Marc Beauregard

**Affiliations:** 1 AgroBioChem Department, Laboratory of Biomass and Green Technologies, University of Liège, Gembloux Agro-Bio Tech, 5030 Gembloux, Belgium; 2 Université du Québec à Trois-Rivières, Centre de Recherche sur les Matériaux Lignocellulosiques, C.P. 500, Trois-Rivières, QC G9A 5H7 Canada; 3 PROTEO, Université Laval, Québec, QC G1V 0A6 Canada; 4 Département de Chimie, Biochimie et Physique, Université du Québec à Trois-Rivières, C.P. 500, Trois-Rivières, QC G9A 5H7 Canada

**Keywords:** FTCM, Carbohydrate-binding module, Fluorescent protein, LCB (lignocellulosic biomass), Cellulose, Hemicellulose, Enzymes

## Abstract

**Background:**

Lignocellulosic biomass will progressively become the main source of carbon for a number of products as the Earth’s oil reservoirs disappear. Technology for conversion of wood fiber into bioproducts (wood biorefining) continues to flourish, and access to reliable methods for monitoring modification of such fibers is becoming an important issue. Recently, we developed a simple, rapid approach for detecting four different types of polymer on the surface of wood fibers. Named fluorescent-tagged carbohydrate-binding module (FTCM), this method is based on the fluorescence signal from carbohydrate-binding modules-based probes designed to recognize specific polymers such as crystalline cellulose, amorphous cellulose, xylan, and mannan.

**Results:**

Here we used FTCM to characterize pulps made from softwood and hardwood that were prepared using Kraft or chemical-thermo-mechanical pulping. Comparison of chemical analysis (NREL protocol) and FTCM revealed that FTCM results were consistent with chemical analysis of the hemicellulose composition of both hardwood and softwood samples. Kraft pulping increased the difference between softwood and hardwood surface mannans, and increased xylan exposure. This suggests that Kraft pulping leads to exposure of xylan after removal of both lignin and mannan. Impact of enzyme cocktails from *Trichoderma reesei* (Celluclast 1.5L) and from *Aspergillus* sp. (Carezyme 1000L) was investigated by analysis of hydrolyzed sugars and by FTCM. Both enzymes preparations released cellobiose and glucose from pulps, with the cocktail from *Trichoderma* being the most efficient. Enzymatic treatments were not as effective at converting chemical-thermomechanical pulps to simple sugars, regardless of wood type. FTCM revealed that amorphous cellulose was the primary target of either enzyme preparation, which resulted in a higher proportion of crystalline cellulose on the surface after enzymatic treatment. FTCM confirmed that enzymes from *Aspergillus* had little impact on exposed hemicelluloses, but that enzymes from the more aggressive *Trichoderma* cocktail reduced hemicelluloses at the surface.

**Conclusions:**

Overall, this study indicates that treatment with enzymes from *Trichoderma* is appropriate for generating crystalline cellulose at fiber surface. Applications such as nanocellulose or composites requiring chemical resistance would benefit from this enzymatic treatment. The milder enzyme mixture from *Aspergillus* allowed for removal of amorphous cellulose while preserving hemicelluloses at fiber surface, which makes this treatment appropriate for new paper products where surface chemical responsiveness is required.

**Electronic supplementary material:**

The online version of this article (10.1186/s13068-017-0980-0) contains supplementary material, which is available to authorized users.

## Background

Global production of biofuels and bioproducts is increasing steadily because such products are greener alternatives to fossil fuels and their derivatives [1–3]. Concomitantly, numerous new products and technologies based on the conversion of biomass have been developed over the last decade [4–9]. Securing sufficient biomass as raw materials is a prerequisite to moving from a petro-chemical to a bio-chemical economy. Using feedstocks to support first-generation biofuel and bioproducts has shown its limits and produces certain undesirable socio-economic and environmental outcomes [10, 11]. The use of lignocellulosic biomass (LCB, including dedicated lignocellulosic crops, agricultural and forestry residues and municipal and industrial wastes) to produce second-generation biofuel and bioproducts would avoid the negative impacts associated with first-generation feedstocks use [12, 13].

Although LCB is a promising, abundant and renewable resource, it is difficult to treat due to its complex structure consisting of cellulose fibrils wrapped in a network of lignin and hemicelluloses. This network, collectively referred to as the lignin-carbohydrate complex, is highly recalcitrant and difficult to modify [8, 14–18]. Consequently, several steps of pretreatments are needed to isolate each of the components before they can be used in value-added applications.

For the production of biofuels based on carbohydrates from LCB, such as bioethanol, the principal goal is the complete hydrolysis of polysaccharide components (mainly cellulose) of the raw material into monomers for subsequent fermentation [18–22]. Utilization of all other lignocellulosic components is not as well developed but is the focus of intensive research efforts [8, 9, 23, 24]. This “integrated biorefinery” concept involves a succession of steps for transforming the entire lignocellulosic biomass into biofuels and bioproducts. This concept has been demonstrated using a variety of physical, chemical and biological treatments [25–27] in a range of configurations [28–31]. Total utilization of LCB will permit commercial exploitation of the entire lignocellulosic biomass in a wide spectrum of bioproducts and bioenergy [5, 32, 33]. In this context, new bioproducts (e.g. biomaterials, biocomposites, biomembranes and biofilms) from previously unused components of LCB are receiving growing interest because they are also biodegradable, produced from a renewable carbon source and can have a wide variety of applications [5, 7, 34–36]. Unlike bioethanol, specific bioproducts based on lignocellulosic fibers do not require complete separation or deconstruction of the raw lignocellulosic polymers. Removal of some specific components or alteration of structural features of fibers leading to modulation of their physical and chemical properties is often sufficient [5, 7, 32, 37–39].

A largely used green process for the removal or alteration of specific structural features of the biomass is the enzymatic hydrolysis or biocatalysis. Enzymes have been used for improving papermaking processes (for fiber cutting action, peeling, delamination, weakening effect, bleaching, refining) [40–42] and also for the deconstruction of lignocellulosic biopolymers [7, 43–51]. Actually, cellulases from *Trichoderma reesei* are subject to many studies and have been used to efficiently hydrolyze cellulose for decades [40, 52]. Enzymes have high selectivity and turnover frequency, permitting processes with high selectivity and increased productivity on a variety of substrates [53]. For example, enzymatic hydrolysis avoids or drastically decreases the production of degradation products that are generated by classical acid hydrolysis (e.g. 5-hydroxymethylfurfural, 2-furfural) [54, 55]. Many types of enzyme can catalyze LCB hydrolysis: endo- and exo-glucanase, cellobiase, xylanase, mannanase and many others. Synergy between several enzymes in a mixture of their lignocellulosic substrates has also been demonstrated, but are not yet completely known [52, 56–58]. In addition to this, enzymes are costly, and accordingly, real-time dosage control is an important parameter in most industrial processes [57, 59–63].

The effectiveness and impact of enzymatic processes on a substrate can be quantified using physical and chemical methods. Among them, the most commonly used are: compositional analysis of the substrate after treatment (using FTIR, XPS) or of the hydrolysates (hydrolysis products content, using GC or HPLC), surface imaging (using SEM, TEM and AFM), index of crystallinity (using XRD and NMR) and mass balance calculations [64–66]. However, current methods of analysis cannot directly monitor enzymatic action. It is not possible to determine the precise order in which components of the substrate were hydrolyzed as the enzymes penetrate the materials and what components are left exposed on fibers after treatment. While direct chemical characterization of the surface is possible with XPS, it remains that this method is expensive and does not distinguish between different polysaccharides because they harbor similar functional groups [67].

The ability to directly monitor changes to the surface of LCB fibers during enzymatic treatment is essential for controlling and optimizing processes according to the final bioproducts targeted. To this end, a rapid and low-cost method to directly monitor the deconstruction of heterogeneous LCB during enzymatic hydrolysis has been developed [67, 68]. Called fluorescent-tagged carbohydrate-binding module method, or FTCM, this method is based on the use of four specific ready-to-use probes made of fluorescent-tagged recombinant carbohydrate-binding modules (named ft-CBM or probes throughout the text). In these probes, the recombinant CBM part binds to a specific component of the substrate surface. The fluorescence of the probe permits rapid quantification of the probes bound to the surface. The fluorescence can be measured by using an ordinary fluorescence plate reader. This new approach allows for specific surface changes to be tracked and for changes to biopolymers, in this case mannan, xylan, crystalline and amorphous cellulose, to be monitored. FTCM can detect these polymers at the surface of the substrate before and after any given treatment, be it mechanical, chemical or enzymatic [67, 68].

In this study, we use FTCM to characterize how the surfaces of a variety of lignocellulosic biomass are modified by two different commercial enzyme cocktails. The substrates include two chemical-thermo-mechanical pulps, referred to as CTM pulps, and two Kraft wood pulps. This investigation provides information on which combination of enzyme treatment and biomass substrate is best suited for industrial applications in which various levels of fiber deconstruction and precise control of fiber surface composition are desirable, such as the production of nanocellulose, fiber-reinforce composites, or paper.

## Methods

### Lignocellulosic biomass

Four wood pulps were selected to evaluate the effect of woody biomass composition and pretreatment on the experiment. Hardwood mix Kraft pulp (here referenced as HK) was kindly provided by Burgo Ardennes S.A. (Virton, Belgium). Softwood from spruce chemical-thermo-mechanical pulp (referenced as SM) and hardwood from poplar chemical-thermo-mechanical pulp (referenced as HM) were kindly provided by SAPPI Lanaken N.V. (Lanaken, Belgium). Softwood mix Kraft pulp (referenced as SK) was kindly provided by Kruger Wayagamac Inc. (Trois-Rivières, Canada). All pulps used in this study were unbleached. The chemical composition of the of the pulps was determined according to the NREL-TP-510-42618 standard method [69]. The length, width, fine percentage and zero span breaking length of wood pulp fibers were analyzed with a fiber quality analyzer (FQA) (LDA02-090 HiRes, OpTest Equipment Inc, Hawkesbury Canada) following the TAPPI T271 om-12 and T231 standard methods.

### Enzyme solutions

Two different commercial enzyme mixtures were used in this study, CelluClast 1.5L (Cat No #C2730) and Carezyme 1000L (Cat No #C2605), which were purchased from Sigma-Aldrich. CelluClast 1.5L (named “T” in this study) is a mixture of fungal hydrolytic enzymes from *T. reesei* and principally consists of two cellobiohydrolases and two endoglucanases, as well as small amounts of other cellulases and also various accessory enzymes which function as hemicellulases [40, 57, 70]. Carezyme 1000L (named “A” in this study) consists of a mixture of several hydrolytic enzymes mixture from *Aspergillus* sp.

Both enzyme mixtures are widely employed for hydrolysis and deconstruction of lignocellulosic biomass. Both enzymes mixtures contain cellulase (CMCase), xylanase, and mannanase enzymes, whose activities were tested using carboxymethyl cellulose, xylan from birch wood, and galactomannan as substrates, respectively. The activities of cellulase, mannanase, and xylanase were assayed quantitatively using the 3,5-dinitrosalicylic acid (DNS) method which measures the reducing sugars generated by enzymatic hydrolysis from their absorption at 540 nm) as described by Miller [64]. Protein content was quantified using the assay developed by Bradford [71].

### Enzymatic treatments of pulp

Three samples of each pulp were prepared in suspension for three different treatments: one without enzyme addition (control sample, called “Std”), a second to which CelluClast 1.5L was added (called “T”), and third to which Carezyme 1000L was added (called “A”). Prior to enzyme addition, each sample was disintegrated in citrate buffer (having a concentration of 0.05 M and pH 4.8) at 1.2% consistency (24 grams of pulp on an oven dry matter basis in 2 L of buffer) with a standard pulp disintegrator and transferred into a 4-L Erlenmeyer flask. Suspensions were pre-heated until 50 °C using a controlled-environment incubator-shaker (New Brunswick Scientific Inc.). Enzyme solutions were then added to a final loading of 1275 mg of enzyme per gram of oven dry pulp. Hydrolysis was carried out in the incubator at 50 °C for 4 h under continuous orbital agitation (150 rpm). Enzymatic hydrolysis was stopped by incubating the pulp on ice for 15 min. Each sample was filtered and filtrate was boiled in a 95 °C water bath for 10 min and kept frozen at − 20 °C until sugars analysis. Filtration of untreated and enzymes treated pulps produced paper sheets, of 60 ± 2 g m^−2^ in basis weight, as per the TAPPI T205 sp-02 standard methodology. The pH was measured before and after enzymatic treatment.

Optimization of hydrolysis conditions, such as duration and enzymes loading, was done on a small scale at high throughput using 96-wells microtiter plates with 3 mm diameter paper discs. After enzymatic digestion of the discs, FTCM test was applied to detect the optimal condition required for enzymes to promote the efficient degradation in cellulose and hemicellulose.

### Handsheet and paper disc preparation

Four different pulps were used for the preparation of handsheets and paper discs. Handsheets of 60 ± 2 g m^−2^ basis weight were prepared as per the TAPPI T205 sp-02 standard. 3-mm paper discs were punched from handsheet [67].

### Construction of recombinant probe expression systems

All carbohydrate-recognition probe genes were inserted into pET11a expression vectors. CBM 372 3a (*Clostridium thermocellum* CipA, NZYTech), CBM15 (*Cellvibrio japonicas*, Z48928), CBM17 (*Clostridium cellulovorans*, U37056), and CBM27 (*Thermotoga maritima*, NP_229032) genes were synthetized by GenScript. The fluorescent protein genes (eGFP, mOrange2, mCherry, and eCFP) were cloned into the *Dra*III and *Bam*HI sites while the CBM genes were introduced into the *Bsr*GI and *Bam*HI sites. All encoding genes were sequenced to ascertain the integrity and fidelity of the probes. The resulting probes GC3a, OC15, CC17, and CC27 [67, 68] were used to detect crystalline cellulose, xylan, amorphous cellulose, and mannan, respectively.

### Expression and purification of probes

All probes were produced in *E. coli* BL21(DE3) Gold pLysS cells and purified as described by Hébert-Ouellet et al. [68].

### Quantification of the carbohydrates on the surface of fiber paper discs using FTCM

Tracking of the variation of carbohydrate on the surface of paper discs using the four different probes was done as described by Khatri et al. and Hébert-Ouellet et al. [67, 68]. Note that lignin fluorescence was subtracted from total fluorescence and that affinity of all probes used here for their respective substrates was previously characterized, as detailed in [67, 68].

### Sugar analysis

After enzymatic hydrolysis, a filtered hydrolysate was analyzed for cellobiose, glucose, xylose, and mannose concentrations using a HPAEC-PAD (Dionex ICS-5000+) and a GC-FID (Agilent Technologies 7890B) following methods from the work of Vanderghem et al. [72, 73]. Results were processed using Chromeleon 7^®^ and OpenLAB CDS ChemStation software.

### Scanning electron microscope (SEM) images

Scanning electron microscope (SEM) images were used to analyze surface morphology and to characterize the effect of the pulping process on paper fibers. Samples of dried handsheets having a basis weight of 60 g ± 2 g m^−2^ were coated with gold in a Quorum SC-7620 sputter-coater. Images were produced of several different locations on the surface of SM and SK pulp samples with a scanning electron microscope (JEOL, JSM-5500).

### Statistical analysis

Minitab 17© and Microsoft Excel 2010© software were used for statistical analysis of data.

## Results and discussion

### Enzyme characterization

Two commercial enzyme mixtures produced by *T. reesei* and by *Aspergillus* sp. were used for this study. Under our specific assay conditions, both commercial preparations contained cellulase (CMCase), xylanase, and moderate mannanase activities. Enzyme mixture T was characterized by higher cellulase and xylanase activities, although its low mannanase activity was roughly equal to mixture A (Additional file 1).

### Pulp fiber characterization

**Fig. 1:**
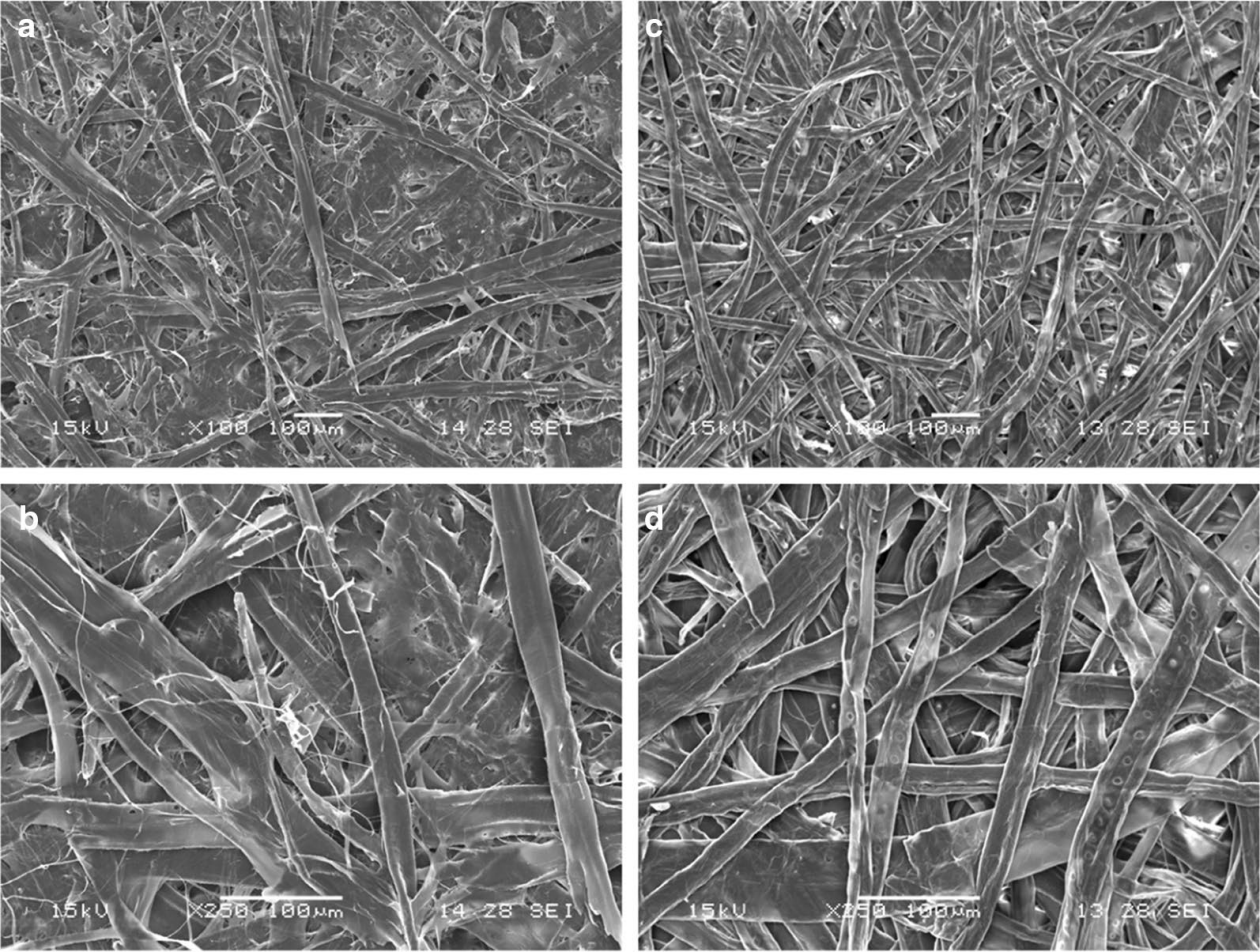
SEM micrographs obtained from (SM) untreated softwood CTM pulp (**a**, **b**), and from (SK) untreated softwood Kraft pulp (**c**, **d**) at two levels of resolution

Pulp fiber characteristics prior to treatments are presented in Table 1, which show how the pulp grades used in this experiment differed from one another. As expected, softwood fibers were longer and wider than hardwood fibers [17]. All of the grades contained similar quantities of fine fibers except for the softwood Kraft pulp. These fine fibers could impair hydrolysis yield on full fibers because finer fibers have a greater susceptibility for hydrolysis, so hydrolysis yield is altered by the quantity of fine fiber in a sample during our 4-h hydrolysis [74]. Hardwood pulp was only slightly affected by Kraft pulping, while for softwood pulp, the Kraft treatment had an obvious impact on length and fines, but none on width. SEM images showed that softwood Kraft pulp has lower fibrillation and greater homogeneity than softwood CTM (Fig. 1) as observed earlier [75, 76] and which is fully compatible with a decreased content in fines.

**Table 1:** Pulp fibers properties before enzymatic treatments

Fibers characteristics (average values)	HM	SM	HK	SK
Length (mm)	0.71	1.31	0.76	2.35
Fines (0–0.2 mm) (%)	15.31	13.25	13.64	3.01
Width (µm)	22.6	27.4	17.7	26.0

*HM* hardwood CTM pulp, *SM* softwood CTM pulp, *HK* hardwood Kraft pulp, and *SK* softwood Kraft pulp

**Fig. 2:**
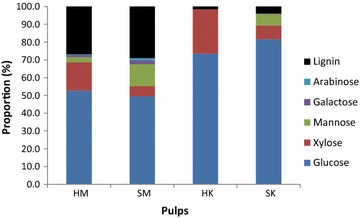
Lignin and carbohydrate monomer content of pulps. *HM* hardwood CTM pulp, *SM* softwood CTM pulp, *HK* hardwood Kraft pulp, and *SK* softwood Kraft pulp

Mechanically treated pulps contained more lignin than the Kraft pulps (Fig. 2). The Kraft process dissolves lignin from wood raw material to liberate fibers, while by contrast mechanical separation of wood fibers does not involve the extraction of lignin [76]. Lignin protects the other components of the biomass against degradation, so the absence of lignin in Kraft pulp permits enzymatic hydrolysis to occur more effectively [77]. As expected, softwood hemicelluloses were glucomannan-rich, while hardwood hemicelluloses were xylose-rich [17, 78, 79]. HK and SK pulps yield the greatest quantity of glucose, making them the most promising of the samples as a potential biofuel substrate.

### Hydrolysate analysis

**Fig. 3:**
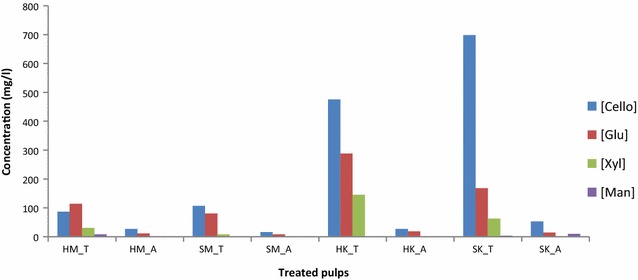
Concentrations of selected carbohydrates in hydrolysate recovered after hydrolysis of pulp. T and A refer to mixtures of enzymes used for hydrolysis

Hydrolysate sugar content of the control samples (i.e. without enzyme addition) was negligible (data not shown). This demonstrates that hydrolysis did not occur in the absence of enzymes. Figure 3 shows cellobiose, glucose, xylose, and mannose concentration of hydrolysate solutions recovered after treating pulps with T and A enzyme cocktails.

Figure 3 presents the amounts of selected mono- and disaccharides which were liberated by enzymatic hydrolysis of pulp fibers. The quantity of sugar detected in the hydrolysate was better related to pulp grade than to the enzyme cocktail used. Kraft pulps released more of each sugar, indicating they are more susceptible to enzymatic hydrolysis in the relevant conditions. This can be explained by the difference in lignin content, since the presence of lignin protects polysaccharides from enzymatic hydrolysis [60, 80–84]. As discussed earlier and in the literature, pretreatments which remove lignin and hemicellulose expose a greater proportion of the cellulose in the substrate and increase pore volume and surface area, which results in increased hydrolysis rate [85]. The high glucose content of the Kraft pulps presented in Fig. 2 suggests that these pulps are composed primarily of cellulose, an inference that is consistent with the composition of the hydrolysate produced from their enzymatic hydrolysis. Hydrolysate sugar content also demonstrated that enzyme “A” was less effective than “T” under same hydrolysis conditions. More xylose was released from hardwood pulp in the presence of T enzyme cocktail, which again corresponds with the abundance of xylose monomers in the substrate, as shown in Fig. 2. The cellobiose yield from hydrolysis of SK was greater than that from hydrolysis of HK, although HK hydrolysis produced more glucose when catalyzed by T treatment. Finally, hydrolysate composition suggests that the mannanase activity of both enzyme cocktails is low. Such results may indicate that mannans are not as accessible as other polymers, or that mannanase activity is too low (consistent with activity measurements for both enzyme preparations; see Additional file 1). The sugar content of the hydrolysates is a good indicator of enzyme activity with respect to specific carbohydrates, but does not provide any information on the surface chemistry of the treated fiber.

### Effect of enzymatic treatment on pulp fibers

Biofuel production from LCB depends on polymer accessibility during enzymatic treatment, but many other applications require specific surface functionality linked to distribution of polymers left after treatment at the surface of fibers. One way to obtain information about the outcome of an enzymatic treatment on LCB is by investigating properties of its fibers and of paper formed using these fibers. Enzyme hydrolysis used here only affected the length of Kraft pulp grade. Treatment of hardwood Kraft pulp with T enzymes decreased length by 20%. Enzymes, A and T, decreased softwood Kraft fiber length by 15 and 25%, respectively (Additional file 2: Figure S1). These results suggest a fiber cutting action, ascribed to endoglucanase activity in enzyme cocktails [57, 70]. While Kraft pulp fiber length decreased as a consequence of treatment, fines increased (Additional file 2: Figure S2). This phenomenon has been suggested as a consequence of the combination of cutting, peeling, delaminating, and weakening effects on the surface of the fibers by enzymatic hydrolysis [40–42]. Although the enzymatic hydrolysis reduced the length of some fibers, it did not affect the average width of any samples, regardless of pulping or enzymes used (Additional file 2: Figure S3). Concerning zero span breaking length, a measure of the average strength of individual fibers (Additional file 2: Figure S4), treatment had no effect on mechanical CTM pulps but both enzymes degraded chemical Kraft pulp strength. The higher lignin content of the mechanical pulps may explain why their mechanical strength was not affected by the treatment. Analysis of these paper properties corroborates previous studies of simple sugars release by hydrolysis of paper pulp and confirms that Kraft pulps are more susceptible to enzymatic treatments [47, 50, 86, 87]. For applications where strength properties are very important, such combination pulp-enzymatic treatments (Kraft pulps treated with cellulase mixtures) would be deleterious.

### Detection of pulp fiber polymers using FTCM analysis before and after enzymatic treatments

**Fig. 4:**
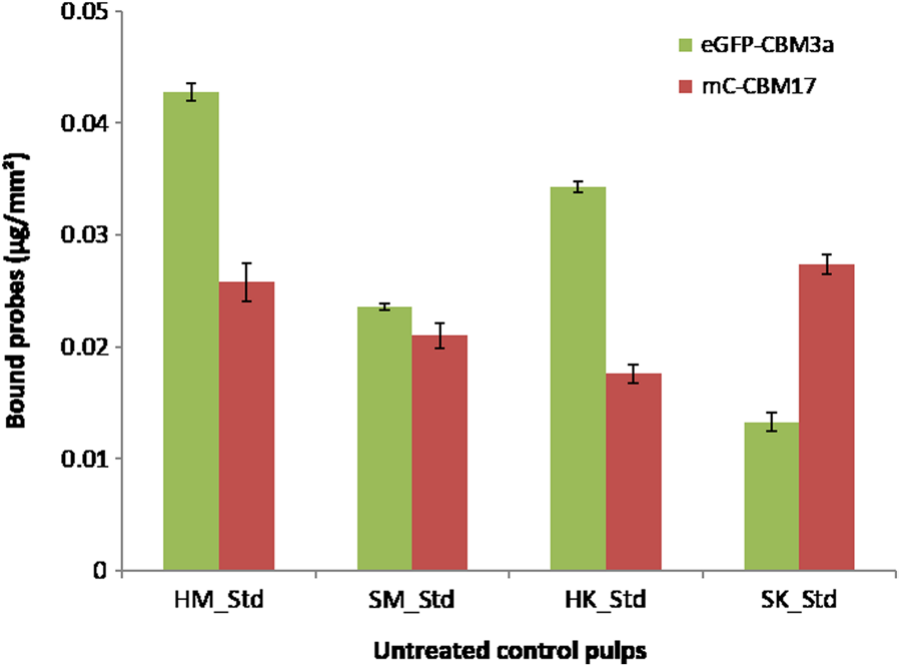
CBM binding to the surface of untreated pulps. The quantity of probe bonded to crystalline cellulose (in green) and amorphous cellulose (in red)

**Fig. 5:**
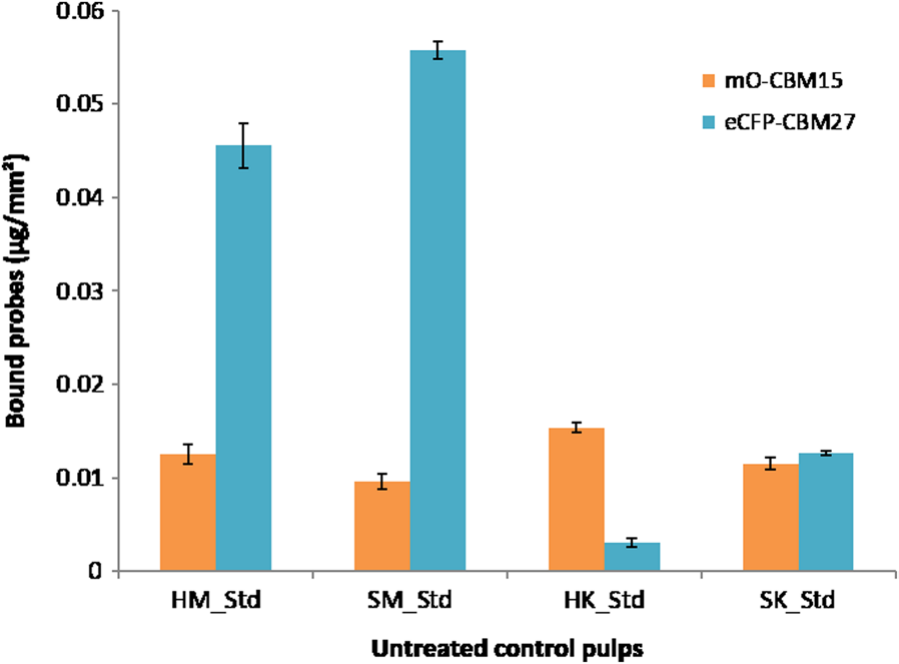
CBM binding to the surface of untreated pulps. The quantity of probe attached to xylan and mannan are shown in orange and cyan, respectively

Fluorescent-tagged carbohydrate-binding module method probes provide a rapid and cost-effective method to map the surface of LCB samples in terms of composition. Running 96 experiments requires a simple plate reader, is currently performed in less than 3 h, and would cost a few dollars when scaled up. Here this analysis was performed using the four probes in order to characterize pulp fibers prior to enzymatic treatments (Figs. 4 and 5). A probe (GC3a) which indicates the presence of crystalline cellulose regions (referred to here as CC) indicated greater CC exposure on hardwood surfaces than on softwood. CC made up a greater proportion of CTM pulps surface than of Kraft pulps surface, despite the higher lignin content of CTM pulps. This result is counterintuitive, since lignin is thought to act as protective barrier around cellulose, but the higher proportion of fibrils and fines in CTM pulps may explain the result since fine fibers tend to have greater specific area and, therefore, offer the most accessible polymers for the probes [68]. Fibrils and fines are partially removed by Kraft pulping, which may explain such results.

Figure 4 also shows the FTCM performed using the amorphous cellulose (referred to as AC) specific probe (named CC17). Mechanical pulps had the strongest AC-binding signal, also in accordance with the explanation of its higher content in high specific surface areas such as fibrils. Although three of the four pulps exposed much less AC than CC, the opposite was observed for SK pulp, where twice as much AC was detected compared to CC. Clearly, the distribution of AC did not parallel CC distribution on the surface of untreated fibers. The total cellulose (CC and AC) detected at the surface was the lowest for SK pulp, where the fibrillations are almost nonexistent as was observed in Fig. 1. This leads to a decrease in high surface area fibrils or fiber fragments, which are primary targets for CBMs binding to fiber polymers. Despite containing more cellulose than CTM pulps, the Kraft pulps returned a weaker binding signal for both CC and AC. Even if the abundance of glucose in the Kraft pulp hydrolysates is consistent with higher cellulose content (Figs. 2 and 3), FTCM shows that CTM pulp fiber surface has a greater number of exposed binding sites for cellulose-specific probes, despite containing less cellulose than Kraft pulps overall. One has to consider that the size of probes used here, with diameters of few nanometers, is closer to water than to most fibrous material. Any probe used here has access to all interstices detectable by electronic microscopy.

OC15 probe, which was used to signal the presence of xylan, returned a more intense signal from untreated hardwood pulps than for softwood (Fig. 5), which is consistent with the previously reported tendency of hardwoods to have a greater xylan content than softwoods [17, 78], and with the monosaccharide content of the samples already shown in Fig. 2. This phenomenon resembles the one observed for CC (Fig. 4), with higher signal for hardwood pulps than for softwood.

The signal produced by the mannan-specific probe (CC27) does not follow the trend described by the probes that have already been described in this section. Mannans were detected in greater abundance on the surfaces of the CTM pulps and were nearly absent from the Kraft pulps. Mechanical pulping of softwoods has been known to partially dissolve mannans [88], but the dearth of mannan on the probe-accessible surface of Kraft pulps suggests that some element of the Kraft process removes mannans even more extensively [89], while by contrast the mechanical treatment leaves them available for probe binding. The disparity in mannan detected on SK and HK corresponds to the relative abundance of mannose contained in the samples as determined in Fig. 2. Comparison of the four pulps’ signals suggests that mannans are strongly associated with lignin. These observations confirm other studies on the lignin–carbohydrate complex organization and changes according to the pulping process [17, 90–93].

**Fig. 6:**
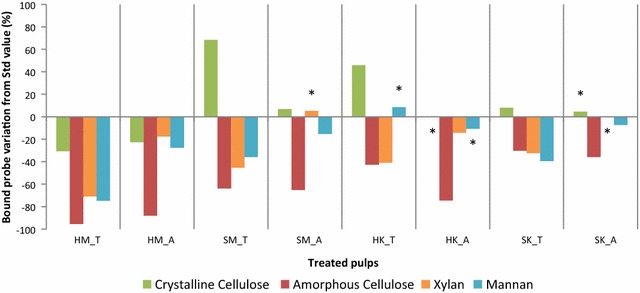
Impact of enzyme (T and A) hydrolysis on the binding of different polymer by probes (crystalline cellulose bound by GC3a, amorphous cellulose bound by CC17, xylan bound by OC15 and mannan by CC27), on the surface of fiber discs. Results that did not deviate significantly from untreated (Std) values (where significance was determined using Dunnet’s comparison test) are indicated with an asterisk (*)

The impact of enzymatic treatments on the amount of each polymer present on the surface of paper discs was characterized using FTCM. In Fig. 6, the signal intensity from each probe is presented in terms of its change relative to the intensity of the corresponding probe on untreated (Std) pulps shown in Figs. 4 and 5. Generally, enzymatic treatments resulted in a decrease in the number of bound probes, although there were some exceptions. This decrease can be a consequence of the preferred degradation of high specific surface components such as fines, filaments and fibrils by enzymes as discussed above. The overall diminution of probe signal intensity may also indicate that the enzymatic treatment results in an increase in the proportion of substances on the substrate surface which are affected neither by the enzymes nor by the probes (e.g. lignin). AC detection invariably decreased after enzymatic treatments, which supports the hypothesis that this component was degraded preferentially by cellulases in both enzymatic cocktails during short-time hydrolysis suggested by several studies [17, 56, 94, 95]. In our assay, changes in AC probe binding did not directly correlate to the yield of hydrolysis products of cellulose (cellobiose and glucose, Fig. 3). Generation of simple sugars such as glucose or cellobiose is a consequence not only of AC but of CC hydrolysis, and the proportions of AC and CC hydrolysis may vary for different pulps and enzyme cocktails.

A general inspection of Fig. 6 reveals that differences in signal intensity from probes bound to the substrate were due to a combination of the disparity in pulp properties and the character of the enzyme cocktails used for their treatment (which both have cellulase, xylanase, and mannanase activity). The results of Fig. 6 show that removal of surface hemicelluloses appeared to be more substantial with T enzymes treatment. This corroborates chromatographic analyses showing higher liberation of xylose and mannose after T enzyme treatment and may be attributed to a superior cellulase and xylanase activities in T enzyme preparation. Also, it can be seen that CTM softwood pulp (SM) responded differently to enzymatic treatments compared to HM. After enzymatic treatment, more CC was detected on the surface of the SM substrate, but less on the surface of the HM substrate. The concurrent increase in CC and decrease in AC indicate that the glucose and cellobiose recovered from the hydrolysate (shown in Fig. 3) are principally the products of AC hydrolysis, as opposed to CC hydrolysis. CC hydrolysis cannot be ruled out, however, since FTCM detects CC probe-binding sites left after treatment. Hydrolysis of first polymers on the surface (including CC) can lead to exposure of previously buried CC.

When treated by A enzymes, the increase in CC at the surface of SM pulp was not as significant as after treatment with T enzymes. AC was decreased with similar efficacy, but other polymers were removed with different intensity. The signal from xylan-binding probes was found to be unaffected at the fiber surface after treatment with A enzymes, while that from mannan-binding probes decreased by 15%. As shown in Fig. 3, no xylose was detected in the hydrolysate from treatment with enzymes A, while the hydrolysate produced by T enzymes cocktail contained some xylose. The absence of xylose in A hydrolysate is consistent with the hypothesis that xylan was not consumed in this treatment, as shown in FTCM results, although xylanase activity was measured in this enzyme cocktail.

Despite major differences in fiber properties and pulping conditions, the proportion of HK-binding sites is modified in a similar way to SM when HK pulp was exposed to enzymatic hydrolysis. More CC was exposed at the surface of HK after T enzyme treatment, despite results on fiber length (Additional file 2) and simple sugar analysis (Fig. 3) that suggest extensive cellulose hydrolysis. Although more CC was exposed on the surface of SM after treatment with T enzymes, this was not accompanied either by fiber length reduction or by substantial hydrolysate sugar yields, which suggests that enzyme treatment was less severe with SM than with HK. The change in CC exposure was limited to 46% for HK (less CC was left on the surface of HK after T enzyme than on SM). Regarding HK pulp, Fig. 6 shows that both AC and xylan decreased on the surface of HK paper discs after either enzymatic treatment, but mannan variations were not significant. These results were suggested by chromatographic analyses but were confirmed by FTCM, which also reveals that CC exposure increased after T treatment, information that cannot be obtained by any other method discussed here.

Enzymatic hydrolysis of SK and HK Kraft pulps occurred in an approximately similar pattern, although both enzymes A and T lead to a smaller change in CC on the fiber surface of SK pulp than on HK pulp. AC decreased after both treatments by about 30%. Hydrolysis with cocktail T leads to a 33% decrease in xylan binding in FCTM but treatment with A enzyme left xylan unchanged. This observation is compatible with the detection of free xylose in the hydrolysate. Mannans were consumed to a greater extent in the softwood pulp. Changes in mannan surface coverage observed by FTCM for SK with T enzymes (a decrease of 40%) were not indicated by hydrolysate analysis, although a decrease in surface polymers does not necessarily lead to simple sugar release if the enzymes involved are also of endo- type. In this case, a drop in relative abundance of mannan at the fiber surface cannot be revealed by a chromatographic analysis of simple sugars but is easily detected using FTCM.

### Surface polymer distribution after enzymatic treatments

Here the quantity of each probe bound to surface is expressed as a percent of the total number of probes detected, removing from our assessment any general change in surface binding or availability for binding (such as the decrease in binding due to loss of high surface fragments in Kraft pulps or change in sheet density as hypothesized earlier [68]). There might be some cross-reactivity among substrates and CBM15 (i.e. OC15 binding mainly to xylan, but having some affinity toward cellulose). We found that the affinity of each probe for its main target surpassed affinity for a similar target by tenfold or more [67, 68].

**Fig. 7:**
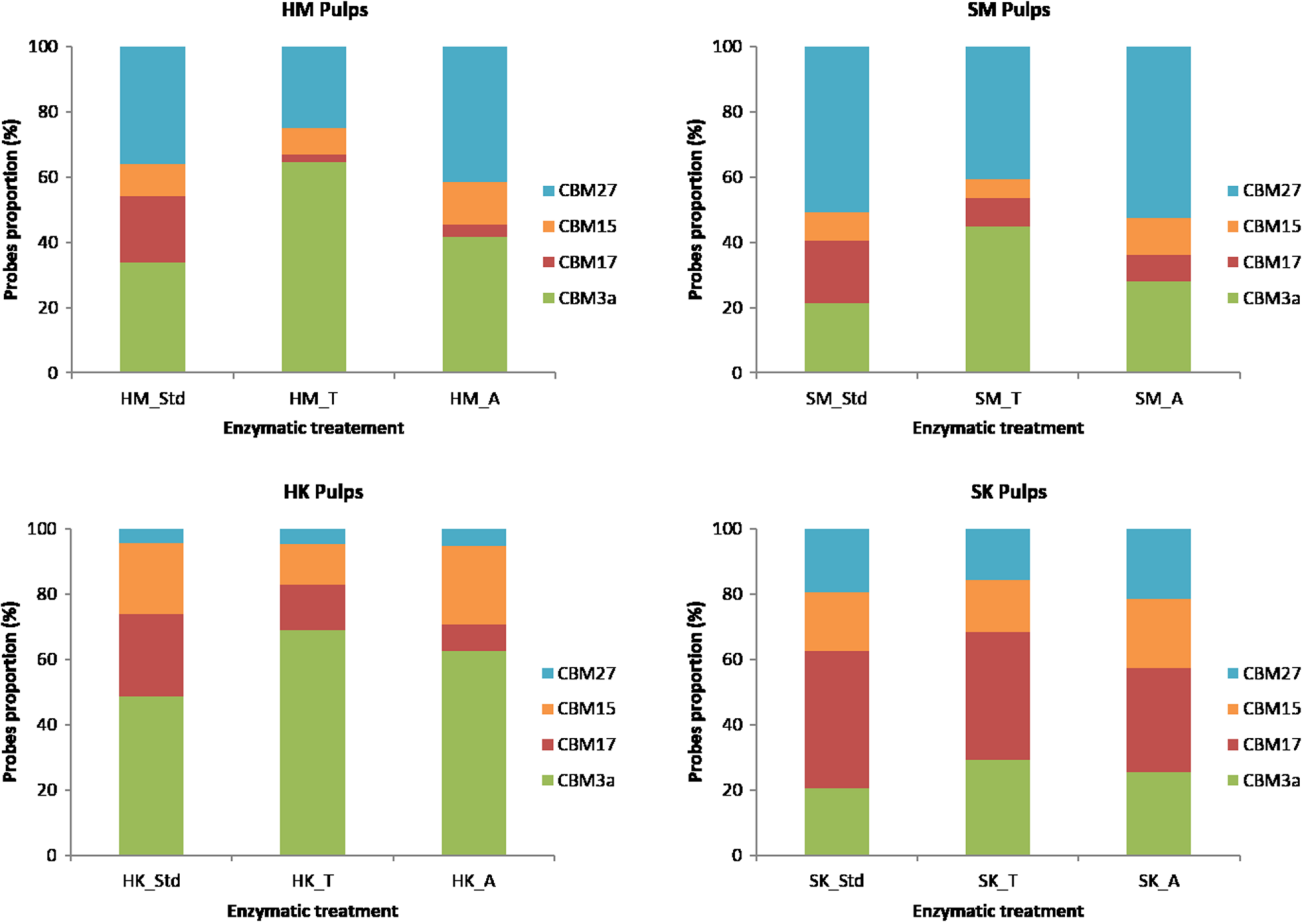
Proportion (in percent) of each probe by treatment on the total probes content for each pulp

The proportions of polymers on the surface of pulps prior to enzymatic treatment are shown in Fig. 7. As expected, given the nature of Kraft pulping, the proportion of AC and CC on the surface of Kraft pulps is higher than in CTM pulps, and although the number of cellulose-binding probes detected on the Kraft pulps surface is less than what was detected on mechanical pulps, a greater proportion of the probes detected on the Kraft pulps were cellulose binding. Also, softwood exposed proportionally more mannan and hardwood more xylan, although the difference between hardwood and softwood was less pronounced for the mechanical pulps. Such distribution of hemicelluloses on the surface is compatible with bulk composition of fibers, and also compatible with the generally accepted understanding of softwood and hardwood hemicellulose composition [17, 78]. In general, CC exposure detection was greater than that of AC regardless of wood or pulping, except for SK pulp, where amorphous regions’ exposure was twice the exposure of CC (the same trend was observed in Fig. 4).

Treatment with enzyme cocktail T consistently left a larger proportion of CC on substrate surfaces, at the expense of AC at the fiber surface. An exception was for SK pulp, where relative amount of AC probe remained stable regardless of enzymatic treatment. SK pulp had the most balanced proportions of probe binding, and this equilibrium between various fractions was barely affected by hydrolysis with T enzyme cocktail. Because analysis of hydrolysates (Fig. 3) revealed a significant release of simple sugars for SK pulp treated with T enzyme, all of the components must have been degraded equally during hydrolysis. Conversely, the relatively small yield of hydrolysate sugars from SK pulp after A enzyme treatment, correlated with nearly same balanced proportion of probe binding, means that SK pulp was not significantly degraded after A enzyme hydrolysis.

Inspection of proportions, and not individual probe binding, allows reconciliation of apparent contradictions between the increase in CC in the SM pulp, shown in Fig. 6, and the low release of sugar after T enzyme treatment (Fig. 3), because the proportion of CC for SM is lower than in HK and HM pulps.

Treatment with enzyme cocktail T results in decreased hemicellulose binding (in proportion to total binding) for all pulps, while treatment with enzyme cocktail A results in probe signal proportions that are in between the control and enzyme T treated substrates. Enzyme A also left larger proportions of hemicelluloses on the surface of fibers at the expense of AC or CC.

The results presented here can be useful in predicting whether an enzymatic treatment of a given biomass is well suited for a given application of wood biomass. For biofuel production, for example, the hydrolysate analysis suggests that best conditions would involve using the most aggressive enzyme (T) with the most exposed fibers (Kraft pulp). Absolute change in probe binding observed by FTCM confirmed the reduction of cellulose at the surface of fibers. FTCM analysis can also be useful for biofuel production, because it can provide precious information about the deconstruction of complex substrates and can monitor the progressive removal of polymers, which permits the optimization of enzymatic treatments. For example, treatment with T enzymes left a higher number of CC-binding sites on all pulps tested here. FTCM would be instrumental in determining the operating conditions which allow for total digestion of CC with minimal costs.

Fluorescent-tagged carbohydrate-binding module method could also provide information for partial hydrolysis of fibers for specific applications. Unlike other methods, such as hydrolysate analysis, chemical analysis, or XPS, FTCM can characterize the surface after treatment. This information can be used to select biomass stock and treatment that will yield the surface properties or composition needed for a given application.

Enzyme T was the most effective for increasing the crystalline cellulose surface proportion and decreasing amorphous cellulose and hemicelluloses. A high production of CC was observed for CTM pulps but Kraft hardwood harbored the highest proportion of CC at surface after treatment. Treatment of HK with T enzymes would be more appropriate for production of purified cellulose products, such as nanocellulose. Treatment with enzyme T would promote generating fiber surfaces that are mechanically stronger, more chemically resistant, and less sensitive to humidity. These characteristics suggest applications like reinforcement in composite materials (in industries like transport, furniture or construction).

Enzyme A is more selective than T. Its use resulted in a significant reduction of the proportion of AC on substrate surfaces while leaving mannan and xylan proportions relatively untouched. This enzyme mixture also hydrolyzed CTM more efficiently than Kraft pulp. Enzyme A allowed the relatively reactive xylan and mannan polymers to be preserved, yielding a product which could be used to develop specialty paper products or insulation materials. The enzymatic treatment of Kraft softwood pulp appears more relevant for applications where an equilibrated distribution of amorphous cellulose and hemicelluloses is preferred. This includes paper products with controlled physical properties, although the strength of these paper products may be decreased by either enzyme.

## Conclusions

Fluorescent-tagged carbohydrate-binding module method can be used as a rapid, affordable, and direct method to evaluate the surface composition of lignocellulosic substrates, thereby permitting processes to be understood in terms of compositional changes on the substrate surface which could not otherwise have been observed. Comparable methods for fiber analysis such as compositional analysis of the substrate after treatment (using FTIR, XPS) or of the hydrolysates (hydrolysis products content, using GC or HPLC), surface imaging (using SEM, TEM, and AFM), index of crystallinity (using XRD and NMR) and mass balance calculations [64–66] cannot directly monitor processing by enzymatic action. The FTCM analysis presented here directly provided valuable information about the quantification of exposed amorphous and crystalline cellulose, xylan, and mannan, which could then be used to determine the effects of pulping and enzymatic hydrolysis on the surface composition of substrates. The variation of these components at surface before and after treatment can guide strategies for preparation of wood fiber derived products.

## Additional files

**Additional file 1: Table S1.** Protein content and activities of the two commercial enzyme mixtures. Enzyme cocktail T refers to CelluClast 1.5L from *Trichoderma reesei* and enzyme cocktail A refers to Carezyme 1000L from *Aspergillus* sp.

**Additional file 2: Figure S1.** Weighted average values of fiber lengths (mm) and standard deviations for control Std, T, and A enzymes treated pulps of different grades. (HM) hardwood CTM pulp; (SM) softwood CTM pulp; (HK) hardwood Kraft pulp, and (SK) softwood Kraft pulp. **Figure S2.** Weighted proportion (%) and standard deviations of fines (fiber with length <0.2 mm) for control Std, T, and A enzymes treated pulps of different grades. (HM) hardwood CTM pulp; (SM) softwood CTM pulp; (HK) hardwood Kraft pulp and (SK) softwood Kraft pulp. **Figure S3.** Arithmetic average values (µm) and standard deviations of fiber widths for control Std, T, and A enzymes treated pulps of different grades. (HM) hardwood CTM pulp; (SM) softwood CTM pulp; (HK) hardwood Kraft pulp, and (SK) softwood Kraft pulp. **Figure S4.** Zero span breaking length (km) for control Std, T, and A enzymes treated pulps of different grades. (HM) hardwood CTM pulp; (SM) softwood CTM pulp; (HK) hardwood Kraft pulp, and (SK) softwood Kraft pulp.

## Abbreviations

AC: amorphous cellulose
AFM: atomic force microscopy
CC: crystalline cellulose
CMCase: carboxymethyl cellulase
CTM: chemical-thermo-mechanical
DNS: 3,5-dinitrosalicylic acid
FQA: fiber quality analyzer
FTCM: fluorescent tagged carbohydrate-binding module method
ft-CBM: fluorescent-tagged recombinant carbohydrate-binding module
FTIR: Fourier transform infrared spectroscopy
GC: gas chromatography
HK: hardwood Kraft pulp
HM: hardwood chemical-thermo-mechanical pulp
HPLC: high-performance liquid chromatography
LCB: lignocellulosic biomass
NMR: nuclear magnetic resonance
NREL: national renewable energy laboratory
SEM: scanning electron microscopy
SK: softwood Kraft pulp
SM: softwood chemical-thermo-mechanical pulp
TAPPI: technical association of the pulp and paper industry
TEM: transmission electron microscopy
XPS: X-ray photoelectron spectrometry
XRD: X-ray diffraction
