# Supplementary material for: Predicting the most appropriate wood biomass for selected industrial applications: comparison of wood, pulping, and enzymatic treatments using fluorescent-tagged carbohydrate-binding modules
Source: Biotechnol Biofuels. 2017 Dec 6;10:293. doi: 10.1186/s13068-017-0980-0 (PMC5718010; doi:10.1186/s13068-017-0980-0)
Supplement: Supplementary file 1 — Additional file 1: Table S1. Protein content and activities of the two commercial enzyme mixtures. Enzyme cocktail T refers to CelluClast 1.5L from Trichoderma reesei and enzyme cocktail A refers to Carezyme 1000L from Aspergillus sp. [file 13068_2017_980_MOESM1_ESM.docx]

**Additional file 1**

Table S1. Protein content and activities of the two commercial enzymes mixtures. Enzymes T refers to CelluClast 1.5L from *Trichoderma reesei* and enzymes A refers to Carezyme 1000L from *Aspergillus* sp.

| Characteristic | Enzymes T | Enzymes A |
| --- | --- | --- |
| Protein content (mg.ml^-1^) | 55,42 | 10,85 |
| CMCase activity (IU.mg^-1^) | 24,70 | 17,87 |
| Xylanase activity (IU.mg^-1^) | 18,95 | 13,76 |
| Mannanase activity (IU.mg^-1^) | 3,94 | 4,17 |
