# Supplementary material for: Predicting the most appropriate wood biomass for selected industrial applications: comparison of wood, pulping, and enzymatic treatments using fluorescent-tagged carbohydrate-binding modules
Source: Biotechnol Biofuels. 2017 Dec 6;10:293. doi: 10.1186/s13068-017-0980-0 (PMC5718010; doi:10.1186/s13068-017-0980-0)
Supplement: Supplementary file 2 — Additional file 2: Figure S1. Weighted average values of fiber lengths (mm) and standard deviations for control Std, T, and A enzymes treated pulps of different grades. (HM) hardwood CTM pulp; (SM) softwood CTM pulp; (HK) hardwood Kraft pulp, and (SK) softwood Kraft pulp. Figure S2. Weighted proportion (%) and standard deviations of fines (fiber with length <0.2 mm) for control Std, T, and A enzymes treated pulps of different grades. (HM) hardwood CTM pulp; (SM) softwood CTM pulp; (HK) hardwood Kraft pulp and (SK) softwood Kraft pulp. Figure S3. Arithmetic average values (µm) and standard deviations of fiber widths for control Std, T, and A enzymes treated pulps of different grades. (HM) hardwood CTM pulp; (SM) softwood CTM pulp; (HK) hardwood Kraft pulp, and (SK) softwood Kraft pulp. Figure S4. Zero span breaking length (km) for control Std, T, and A enzymes treated pulps of different grades. (HM) hardwood CTM pulp; (SM) softwood CTM pulp; (HK) hardwood Kraft pulp, and (SK) softwood Kraft pulp. [file 13068_2017_980_MOESM2_ESM.docx]

**Additional file 2**

Figure S1. Weighted average values of fiber lengths (mm) and standard deviations for control Std, T and A enzymes treated pulps of different grades. (HM) hardwood CTM pulp; (SM) softwood CTM pulp; (HK) hardwood Kraft pulp and (SK) softwood Kraft pulp.

Figure S2. Weighted proportion (%) and standard deviations of fines (fiber with length <0,2 mm) for control Std, T and A enzymes treated pulps of different grades. (HM) hardwood CTM pulp; (SM) softwood CTM pulp; (HK) hardwood Kraft pulp and (SK) softwood Kraft pulp.

Figure S3. Arithmetic average values (µm) and standard deviations of fiber widths for control Std, T and A enzymes treated pulps of different grades. (HM) hardwood CTM pulp; (SM) softwood CTM pulp; (HK) hardwood Kraft pulp and (SK) softwood Kraft pulp.

Figure S4. Zero span breaking length (km) for control Std, T and A enzymes treated pulps of different grades. (HM) hardwood CTM pulp; (SM) softwood CTM pulp; (HK) hardwood Kraft pulp and (SK) softwood Kraft pulp.
